# Outcomes of patients with Juvenile Polyposis-Hereditary Haemorrhagic Telangiectasia caused by pathogenic *SMAD4* variants in a pan-Scotland cohort

**DOI:** 10.1038/s41431-024-01607-w

**Published:** 2024-04-16

**Authors:** Madeline Pearson, Ruth McGowan, Philip Greene, Wayne Lam, Zofia Miedzybrodzka, Jonathan Berg

**Affiliations:** 1 School of Medicine, University of Dundee, Dundee, Scotland UK; 2 West of Scotland Centre for Genomic Medicine, Glasgow, Scotland UK; 3 South East of Scotland Clinical Genetics Service, Edinburgh, UK; 4 School of Medicine, Medical Sciences, Nutrition and Dentistry, University of Aberdeen, Aberdeen, UK

**Keywords:** Colorectal cancer, Outcomes research, Prognosis, Cancer genetics, Cancer genetics

## Abstract

Constitutional loss of *SMAD4* function results in Juvenile Polyposis-Hereditary Haemorrhagic Telangiectasia Overlap Syndrome (JP-HHT). A retrospective multi-centre case-note review identified 28 patients with a pathogenic *SMAD4* variant from 13 families across all Scottish Clinical Genetics Centres. This provided a complete clinical picture of the Scottish JP-HHT cohort. Colonic polyps were identified in 87% (23/28) and gastric polyps in 67% (12/18) of screened patients. Complication rates were high: 43% (10/23) of patients with polyps required a colectomy and 42% (5/12) required a gastrectomy. Colorectal cancer occurred in 25% (7/28) of patients, at a median age of 33 years. Pulmonary arteriovenous malformations were identified in 42% (8/19) of screened patients. 88% (23/26) and 81% (17/21) of patients exhibited JP and HHT features respectively, with 70% (14/20) demonstrating features of both conditions. We have shown that individuals with a pathogenic *SMAD4* variant are all at high risk of both gastrointestinal neoplasia and HHT-related vascular complications, requiring a comprehensive screening programme.

## Introduction

Juvenile Polyposis – Hereditary Haemorrhagic Telangiectasia overlap syndrome (JP-HHT OMIM175050) was first observed in 1980 [1], although its genetic cause – heterozygous pathogenic *SMAD4* loss of function variants [2] – was only identified in 2004 [2]. SMAD4 is the common SMAD protein involved in SMAD mediated downstream signalling of all serine threonine kinase receptors [3, 4]. The combined phenotype occurs because SMAD4 mediates signalling through the BMPR1A receptor implicated in juvenile polyposis (JP, OMIM174900), and ACVRL1/Endoglin signalling involved in Hereditary Haemorrhagic Telangiectasia (HHT, OMIM187300 &600376). In addition, SMAD4 mediates signalling through the Transforming Growth Factor Beta Receptor signalling pathway implicated in Loeys-Dietz syndrome (LDS).

JP is characterised by the presence of multiple gastrointestinal hamartomatous polyps, primarily within the colon. Polyps may develop in childhood, presenting most commonly with rectal bleeding, intussusception, anaemia, or abdominal pain in early adulthood. JP carries a significantly increased risk of colorectal (CRC) and gastric cancer (GC), requiring life-long endoscopic screening [5]. Causal *BMPR1a* or *SMAD4* variants are identified in half of patients [6].

HHT is an autosomal dominant vascular dysplasia, characterised by mucocutaneous telangiectases, epistaxis and visceral arteriovenous malformations (AVMs), particularly affecting the lungs, brain, and liver. *SMAD4* variants are identified in a minority of HHT cases; causal variants are most commonly found in *ENG* or *ACVRL1* [7], affecting around 1 in 5000 people [8].

There are no established diagnostic criteria specific for JP-HHT overlap syndrome; genetic testing supports clinical diagnoses of JP and HHT as individual diseases [9, 10].

A clear understanding of outcomes in pathogenic *SMAD4* variant carriers is essential to inform clinical screening and management. We present data from all patients known to have a pathogenic *SMAD4* variant in Scotland linked to screening data for all 3 clinical phenotypes: HHT, JP and LDS.

## Methods

A retrospective multi-centre case-note review of patients with *SMAD4* loss of function variants was performed. Constitutional *SMAD4* pathogenic variant carriers were identified from all four Scottish clinical genetics centres, and patients with Myhre Syndrome were excluded. Caldicott guardian approval was obtained to allow data access at each site. A total of 28 patients from 13 families across Scotland were identified. Information on clinical features of JP, HHT and LDS were collected along with screening outcomes. No uniform management guidelines were followed. A literature review identified 261 patients with JP-HHT from 48 papers (Supplementary Information).

## Results

**Fig. 1:**
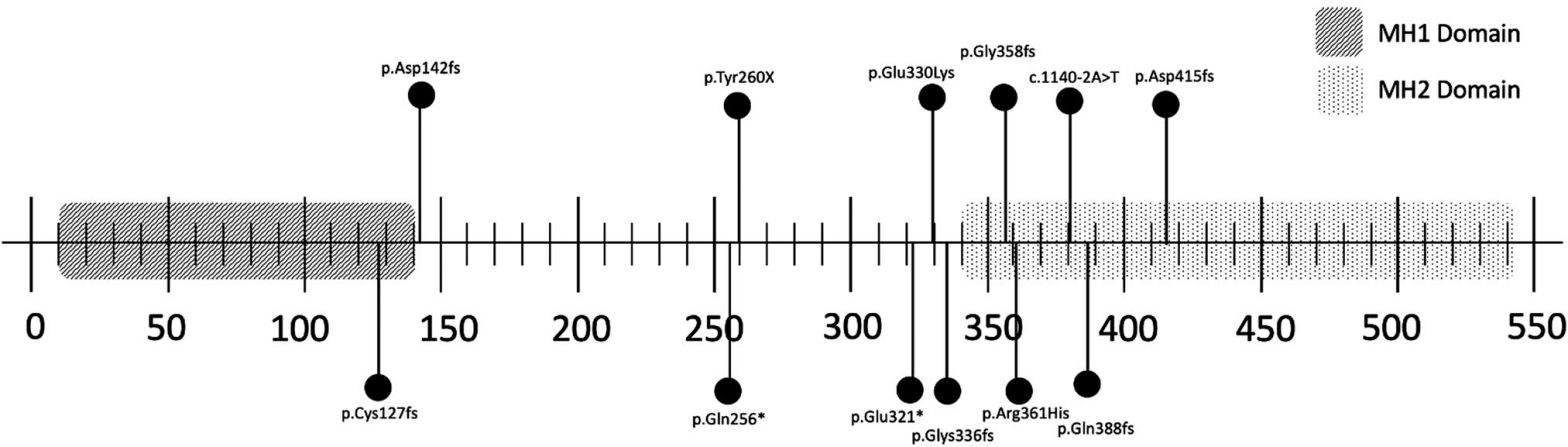
*SMAD4* pathogenic variants in the Scottish Cohort of JP-HHT patients. The mad homology 1 domain (MH1) at the amino-terminal and mad homology 2 domain (MH2) at the carboxy-terminus are highlighted with stripes and dots, respectively. The sites of the pathogenic variants in the Scottish cohort of JP-HHT patients are marked. In addition, one patient had a whole *SMAD4* gene deletion. All variants were reported by an International Organisation for Standardisation accredited laboratory.

Thirteen families with JP-HHT were identified, totalling 28 patients. Each family had a unique pathogenic variant (Fig. 1). 39% were male (*n* = 11), with 61% female (*n* = 17). Median age at the study-time was 37.0 years (IQR 28.8–45.5). Summated clinical features are compared to previously reported cases (Supplementary Information) in Table 1. A comparison of clinical features between index cases and cascade-identified individuals is given in Table 2.

**Table 1:** Clinical features in the Scottish Juvenile-Polyposis Hereditary Haemorrhagic Telangiectasia Cohort.

				Scottish Cohort		Literature	
				*N* = 28	(%)	*N* = 261	(%)
General	Sex (M:F)			11:17			
	Age, median (IQR)			37	(29–46)		
Juvenile Polyposis Features Features	Colonic Polyps		Screened	23/28	(82)	181/193	(94)
			Present	20/23	(87)		
	Upper Gastrointestinal Polyps	Gastric Polyps	Screened	18/28	(64)	113/171	(66)
			Present	12/18	(67)		
		Small Bowel Polyps	Screened	9/28	(32)		
			Present	7/9	(78)		
	Colectomy			10/28	(36)	56/193	(29)
	Gastrectomy			5/28	(18)	33/171	(19)
	Colorectal cancer			7/28	(25)	22/149	(15)
	Gastric cancer			1/28	(4)	6/101	(6)
Hereditary Haemorrhagic Telangiectasia Features	Epistaxis		Screened	20/28	(71)	163/208	(78)
			Present	15/20	(75)		
	Telangiectasia		Screened	14/28	(50)	121/198	(61)
			Present	4/14	(29)		
	Pulmonary AVM		Screened	19/28	(68)	110/178	(62)
			Present	8/19	(42)		
	Cerebral AVM		Screened	2/28	(7)	21/153	(14)
			Present	0/2	(0)		
	Hepatic AVM		Screened	2/28	(7)	32/119	(27)
			Present	1/2	(50)		
	Stroke			2/28	(7)	10/102	(10)
Other Features	Thoracic Artery Aneurysm		Screened	4/28	(14)	22/133	(17)
			Present	0/4	(0)		
	Anaemia			15/28	(54)	85/138	(62)

The screening uptake and clinical features of JP, HHT and LDS that were identified in the Scottish cohort are reported. Where the data was available in the literature, the number of patients who were screened is shown.

**Table 2:** Clinical Features in the Scottish Juvenile-Polyposis Hereditary Haemorrhagic Telangiectasia Cohort separated by proband and cascade-identified.

				Proband		Cascade Identified	
				*N* = 13	(%)	*N* = 15	(%)
General	Sex (M:F)			8:5		6:5	
	Age, median (IQR)			36	(28–40)	39	(32–62)
Juvenile Polyposis Features	Colonic Polyps		Screened	13/13	(100)	10/15	(67)
			Present	11/13	(85)	9/10	(90)
	Upper Gastrointestinal Polyps	Gastric Polyps	Screened	11/13	(85)	7/15	(47)
			Present	6/11	(55)	6/7	(86)
		Small Bowel Polyps	Screened	4/13	(31)	5/15	(33)
			Present	3/4	(75)	4/5	(80)
	Colectomy			5/13	(38)	5/15	(33)
	Gastrectomy			3/13	(23)	2/15	(13)
	Colorectal cancer			2/13	(15)	5/15	(33)
	Gastric cancer			0/13	(0)	1/15	(7)
Hereditary Haemorrhagic Telangiectasia Features	Epistaxis		Screened	11/13	(85)	9/15	(60)
			Present	9/11	(82)	6/9	(67)
	Telangiectasia		Screened	8/13	(62)	5/15	(40)
			Present	3/8	(38)	1/6	(17)
	Pulmonary AVM		Screened	11/13	(85)	8/15	(53)
			Present	5/11	(45)	3/8	(38)

The clinical features of JP, HHT and LDS identified in the Scottish cohort are shown, separated by proband and cascade-identified patients.

### Features of juvenile polyposis syndrome

82% (23/28) of the cohort had colonic screening, with colonic polyps identified in 87% of those screened (20/23). The median age for initial identification of colonic polyps was 24.0 years (IQR 21.0–33.8) (*n* = 16). The age at identification of colonic polyps was similar between probands and cascade-identified individuals (21.0 years vs 25.0 years, *p* = 0.25). 43% had a colectomy (10/23), at a median age of 24.0 years (IQR 20.0–47.0) (*n* = 9). Colectomy indications included bowel cancer (*n* = 4), extensive polyps unsuitable for resection (*n* = 4), bowel perforation (*n* = 1) and intussusception (*n* = 1). Screening of the upper gastrointestinal tract—stomach or small intestine—occurred in 64% (18/28) and 32% (9/28) of patients respectively. Small bowel polyps were identified in 78% of those screened (7/9) in the duodenum (*n* = 4), jejunum (*n* = 1), ileum (*n* = 1) and unspecified (*n* = 1), and gastric polyposis was seen in 67% (12/18). 18% of the total cohort (5/28) underwent a gastrectomy although in those with known gastric polyposis the gastrectomy risk was 42% (5/12). Gastrectomy indications were obstructive symptoms (*n* = 3) or bleeding (*n* = 2). The median age at gastrectomy was 47.0 years (IQR 37.0–59.0) (*n* = 5).

A history of CRC was present in 25% of patients (7/28), diagnosed at a median age of 33 years (29.5–45.0) (*n* = 7). This was fatal in 3 patients. All CRC cases were diagnosed at, or before, the time of *SMAD4* variant identification; none arose during surveillance. Only one patient was diagnosed with gastric cancer, at age 66 years.

### Features of hereditary haemorrhagic telangiectasia

A total of 71% of patients were asked about epistaxis (20/28) of which 75% reported its presence (15/20). When an onset age was documented, this was universally in childhood (*n* = 8). Examination for cutaneous telangiectasia was formally documented in 50% of patients (14/28); 29% of whom had lesions (4/14).

Sixty eight percent of patients underwent pulmonary AVM (PAVM) screening (19/28) via transthoracic echocardiography (*n* = 10) and/or computerised tomography pulmonary angiogram (*n* = 12). A PAVM was identified in 47% (9/19) at a median age of 25.5 years (IQR 17.5–32.8) (*n* = 5). 56% of PAVMs required intervention (5/9), at a median age of 30.5 years (IQR 15.8–46.3) (*n* = 3). Indicators of an underlying PAVM included digital clubbing in 11% (3/28), migraines in 25% (7/28) and exercise intolerance in 14% (4/28). A stroke occurred in two patients, one of whom had a PAVM.

Screening for other AVMs was limited. One gastrointestinal and one renal AVM were detected in our cohort. Two patients had negative screening for brain AVMs. One patient had multiple small hepatic AVMs.

### Other features

Anaemia was documented in 54% of patients (15/28). Only 14% of patients received screening for a thoracic aortic aneurysm (4/28), all of whom were negative. Other vascular abnormalities were documented in 2 patients (7%); one patient had a renal artery aneurysm, and another had a complex history of recurrent thrombosis. One patient had joint hypermobility, but none had other connective tissue disease features, such as valvular regurgitation or retinal detachment, documented. Two patients (7%) had pectus excavatum.

Overall 88% (23/26) of patients with some GI screening had features of JP. Vascular HHT features were present in 81% (17/21) of screened patients. 70% (14/20) of patients who had received screening for both conditions had features of both JP and HHT. Only two patients were non-penetrant for either condition. One patient was too young for screening (age 5 years); the other had no features present at age 41 years. Cascade-identified patients were still at very high risk for actionable complications including GI neoplasia and PAVMs.

## Discussion

*SMAD4* constitutional pathogenic variant carriers comprise a very high-risk, and complex to manage, patient cohort. We found that the vast majority (70%) of constitutional LOF *SMAD4* carriers exhibit features of both JP and HHT; all carriers must receive adequate screening for complications of both conditions. The UK Cancer Genetics Group has recently published a summary of management guidelines for these complex patients [11].

*SMAD4* patients harbour a high risk of early-onset bowel cancer (25% at a median age of 33 years), which proved fatal in almost half of patients. This may be skewed by an early cancer diagnosis leading to *SMAD4* analysis. We have found a higher incidence and earlier onset of cancer than previously reported (11.8% at a median age of 38 years) [12], and, with a median cohort age of 37 years, many of our patients face significant future cancer risks. Bowel complications were not limited to cancer; our cohort also exhibited bowel perforation (related to treatments rather than the primary disease process), intussusception, bowel obstruction and gastric outlet obstruction. We reported a similar rate of GC in our cohort compared to the literature (4% vs 6%). This is surprising due to the cohort age; GC in JP is considered a later feature of disease [12].

The gastrointestinal features of *SMAD4* patients are well recognised. In contrast, the significance of the HHT phenotype has been less documented in previous work. As most JP-HHT patients present along the bowel cancer pathway significant HHT manifestations may be missed. In our cohort only 1 patient received their genetic testing secondary to a HHT diagnosis as compared to JP. However, we show that HHT features can be elicited from the vast majority (81%) of patients. The importance of thorough HHT screening in *SMAD4* patients cannot be understated; systematic screening, with treatment when required, mitigates the reduced life expectancy associated with HHT [13]. In our cohort 31% (8/26) had a documented PAVM, lower than previously reported [14], but still high and in line with PAVM frequency seen in HHT caused by endoglin variants [15]. Many AVMs are likely to remain undiagnosed due to inadequate screening. Our data suggests that HHT features are not just seen in a subset of *SMAD4* patients, they are an integral part of the *SMAD4* syndrome and must be screened for accordingly.

The question of extending screening to include aortic evaluation has been raised [16]. In Scotland the rates of aortic screening remain low (14%) and, as such, it is difficult to estimate the prevalence of this feature.

The phenotype is highly penetrant; our data suggests that disease burden is largely similar between probands and cascade-identified family members. In a cascade-identified individual the presence of a pathogenic variant alone is sufficient to recommend life-long JP-HHT screening. However screening uptake is higher in probands, highlighting an area of unmet need.

Our work suggests that many JP-HHT patients in Scotland remain undiagnosed. The incidence of JP-HHT has been estimated as 1:16,000–1:100,000 [17], compared to the 1:195,000 in our cohort with identified pathogenic variants. A higher index of suspicion for JP-HHT, and increased genetic testing, will improve identification of these high-risk individuals who may not be receiving adequate care.

### Supplementary information

Supplementary Table 1

Supplementary Figure 1

Supplementary Table 2

## Data Availability

Individual patient data from this study is confidential, and therefore cannot be provided. Tabulated summated data is provided with Caldicott guardian approval and contained within the data tables of the paper.
