# Supplementary material for: Outcomes of patients with Juvenile Polyposis-Hereditary Haemorrhagic Telangiectasia caused by pathogenic SMAD4 variants in a pan-Scotland cohort
Source: Eur J Hum Genet. 2024 Apr 16;32(6):731–5. doi: 10.1038/s41431-024-01607-w (PMC11153582; doi:10.1038/s41431-024-01607-w)
Supplement: Supplementary file 1 — Supplementary Table 1 [file 41431_2024_1607_MOESM1_ESM.pdf]

Table 1: Search terms for Juvenile Polyposis-Hereditary Haemorrhagic Telangiectasia Literature Review

Search Number	Search Words	PubMed	WebofScience
S1	SMAD4	5,573	5,574
S2	“SMAD 4”	202	214
S3	MADH4	68	99
S4	“MADH 4”	0	0
S5	S1 OR S2 AOR S3 OR S4	5,695	5,782
S6	“juvenile polyposis”	691	1079
S7	JP	452,729	1, 055, 131
S8	JPS	2,788	8,502
S9	S6 OR S7 OR S8	455,199	1, 063, 011
S10	“hereditary hemorrhagic telangiectasia”	4,177	3,538
S11	“hereditary haemorrhagic telangiectasia”	597	623
S12	HHT	2,501	5,574
S13	S10 OR S11 OR S12	5,305	8,109
S14	S5 AND S9 AND S13	71	95
S15	JP-HHT	14	15
S16	JPS-HHT	7	4
S17	S15 OR S16	21	19
S18	S14 OR S17	72	97
**Database Search Limits Used**			
	By English Language	66	97
