# Supplementary figures and images for: Outcomes of patients with Juvenile Polyposis-Hereditary Haemorrhagic Telangiectasia caused by pathogenic SMAD4 variants in a pan-Scotland cohort
Source: Eur J Hum Genet. 2024 Apr 16;32(6):731–5. doi: 10.1038/s41431-024-01607-w (PMC11153582; doi:10.1038/s41431-024-01607-w)

Figure 1: Flow diagram for identification of JP-HHT cases from the literature

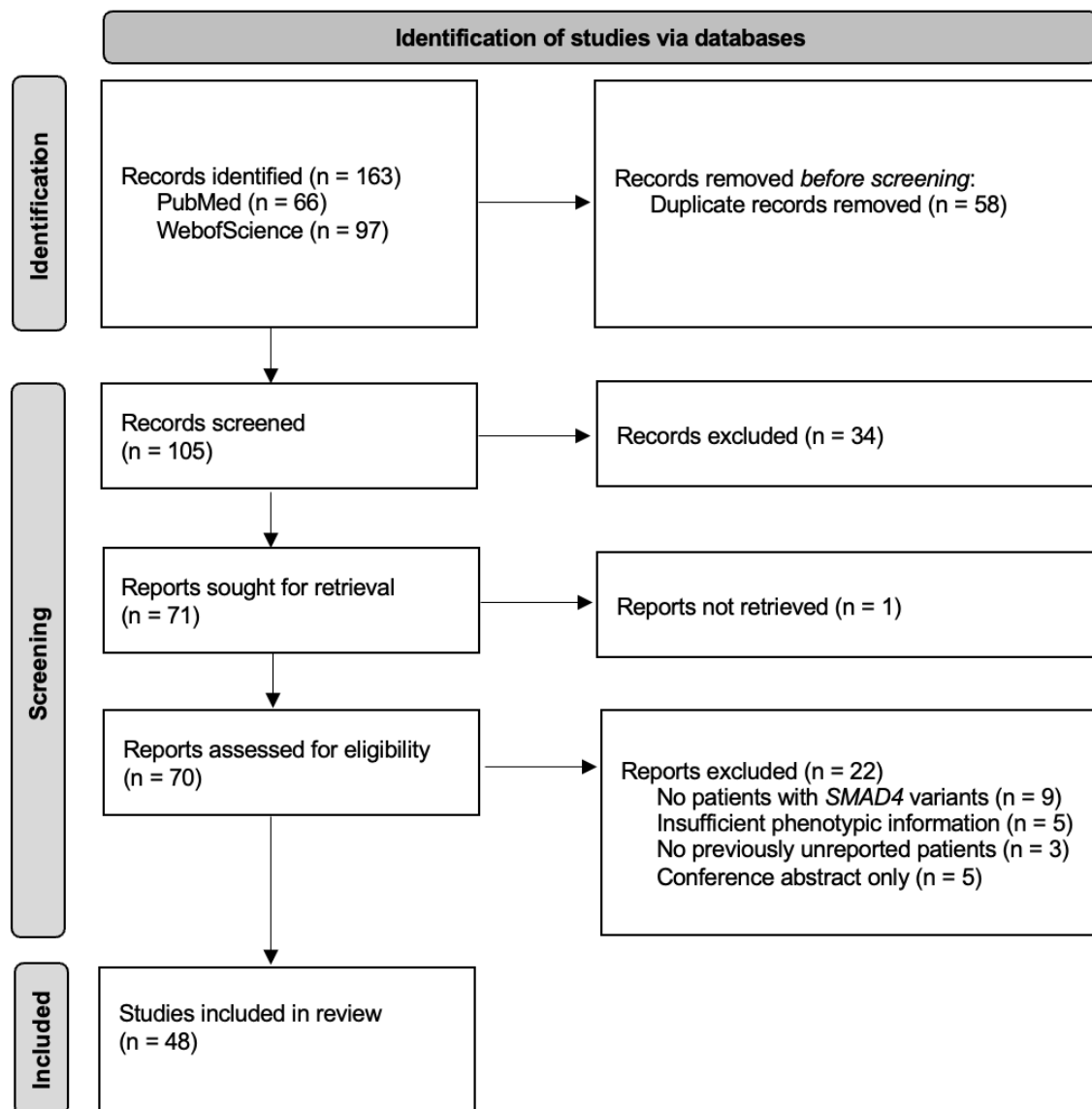

Supplement: Supplementary file 2 — Supplementary Figure 1 [file 41431_2024_1607_MOESM2_ESM.pdf]
