# Supplementary material for: Outcomes of patients with Juvenile Polyposis-Hereditary Haemorrhagic Telangiectasia caused by pathogenic SMAD4 variants in a pan-Scotland cohort
Source: Eur J Hum Genet. 2024 Apr 16;32(6):731–5. doi: 10.1038/s41431-024-01607-w (PMC11153582; doi:10.1038/s41431-024-01607-w)
Supplement: Supplementary file 3 — Supplementary Table 2 [file 41431_2024_1607_MOESM3_ESM.pdf]

Supplementary Table 2: Literature Review of *SMAD4* Juvenile-Polyposis Hereditary Haemorrhagic Telangiectasia Patients

**Paper**		**Mutation**	**Juvenile Polyposis**						**Hereditary Haemorrhagic Telangiectasia**										**Connective Tissue Disease**	
											Visceral Vascular Malformation									
		Documented Pathogenic Mutation	Colonic Polyps	Upper GI Polyps	Colorectal Cancer	Gastric Cancer	Colectomy	Gastrectomy	Epistaxis	Telangiectasia	Pulmonary	Cerebral	Hepatic	Other	Anaemia	Cyanosis	Stroke	Digital Clubbing	Aortopathy***	Valvular Regurgitation
**Gallione** et. al (2004) (1)	*n = 14*	14/14 (100)	12/13 (92)	3/14* (21)	4/13 (31)	-	3/13 (23)	0/14* (0)	9/14 (64)	9/14 (64)	7/11 (64)	4/8** (50)	4/8 (50)	-	12/14 (86)	1/14 (7)	2/14 (14)	7/14 (50)	-	-
**Gallione** et. al (2006) (2)	*n = 3*	3/3 (100)	2/2 (100)	-	1/3 (33)	-	0/2 (0)	-	3/3 (100)	3/3 (100)	2/3 (67)	-	1/3 (33)	1/3 (33)	1/3 (33)	-	-	-	-	-
**Poletto** et. al (2009) (3)	*n = 3*	3/3 (100)	2/3 (67)	1/3 (33)	-	-	0/3 (0)	0/3 (0)	3/3 (100)	1/2 (50)	1/1 (100)	0/1 (0)	-	-	-	-	-	1/3 (33)	-	-
**Gallione** et. al (2010) (4)	*n = 19**	15/19* (79)	18/19* (95)	4/19* (21)	3/19* (16)	-	0/19* (0)	0/19* (0)	17/19 (89)	14/19 (74)	16/19* (84)	1/19* (5)	1/19* (5)	-	10/19 (53)	1/19 (5)	2/19 (11)	7/19 (37)	-	1/19* (5)
**Andrabi** et. al (2011) (5)	*n = 5*	3/5 (60)	5/5 (100)	0/1 (0)	1/5 (20)	-	1/5 (20)	0/1 (0)	1/5* (20)	-	0/1 (0)	0/1 (0)	0/1 (0)	-	-	-	-	-	3/5 (60)	-
**Schwenter** et. al (2012) (6)	n = 3	3/3 (100)	3/3 (100)	1/3 (33)	1/3 (33)	-	1/3 (33)	0/3 (0)	3/3 (100)	0/1 (0)	2/3 (67)	0/3 (0)	-	-	1/3 (33)		-	-	-	3/5 (60)
**Schwenter** et. al (2012) (7)	*n = 14*	13/14 (93)	12/12 (100)	7/10 (70)	3/12 (25)	-	2/12 (17)	0/10 (0)	11/13 (85)	11/13 (85)	8/14 (57)	1/14* (7)	1/14* (7)	-	9/14 (64)	4/13 (31)	2/13 (15)	2/13 (15)	-	8/14* (57)
**O'Malley** et. al (2012) (8)	*n = 21*	21/21 (100)	20/20 (100)	19/19 (100)	-	-	12/20 (60)	8/19 (42)	15/21 (71)	12/21 (57)	15/16 (94)	6/17 (35)	0/1 (0)	3/21 (14)	-	-	-	-	-	-
**Wain** et. al (2014) (9)	*n = 34*	34/34 (100)	31/32 (97)	21/34* (62)	3/32 (9)	0/34 (0)	10/32 (31)	7/34* (21)	19/31 (61)	15/31 (48)	9/17 (53)	1/26 (4)	6/16 (38)	-	24/34 (71)	-	-	3/34 (9)	4/34 (12)	2/34 (6)
**Heald** et. al (2015) (10)	*n = 16*	16/16 (100)	-	-	-	-	-	-	2/5 (40)	2/4 (50)	4/6 (67)	1/6 (17)	-	1/16 (6)	-	-	-	-	6/16 (38)	-
**Aytac** et. al (2015) (11)	*n = 27*	27/27 (100)	25 / 26 (96)	23/26 (88)	-	2/27 (7)	15/26 (58)	7/26 (27)	-	-	-	-	-	-	-	-	-	-	-	-
**De Leon** et. al (2018) (12)	*n = 3*	3/3 (100)	3/3 (100)	3/3 (100)	0/3 (0)	1/3 (33)	0/3 (0)	2/3 (67)	3/3 (100)	0/3 (0)	1/3 (33)	-	-	1/3 (33)	2/3 (67)	-	-	-	-	-
**Kilian** et. al (2020) (13)	*n = 11*	11/11 (100)	-	-	-	-	-	-	10/11 (91)	4/11 (36)	1/11 (9)	0/11 (0)	0/11 (0)	-	2/11 (18)	-	-	-	-	-
**McDonald** et. al (2020) (14)	*n = 22**	22/22* (100)	19/22* (86)	15/22* (68)	2/22* (9)	-	0/22* (0)	0/22* (0)	17/22 (77)	12/22 (55)	10/22* (45)	2/22* (9)	6/22* (27)	2/22* (9)	-	-	1/22 (5)	-	1/22* (5)	-
**Jelsig** et. al (2022) (15)	*n = 29*	29/29 (100)	-	-	-	-	-	-	25/29 (86)	24/29 (83)	12/27 (44)	0/7 (0)	6/9 (67)	-	-	-	-	-	3/22 (14)	-
**Kananazawa** et. al (2022)	*n = 3*	2/3 (67)	3/3 (100)	3/3 (100)	3/3 (100)	1/3 (33)	0/3 (0)	1/3 (33)	3/3 (100)	0/1 (0)	2/3 (67)	-	-	-	2/3 (67)	-	-	-	-	-
**Small Cohorts******	*n = 34**	31 / 34 (91)	26/30 (87)	13/14 (93)	3/34* (9)	2/33 (6)	12/30 (40)	8/14 (57)	22/23 (96)	14/24 (58)	20/21 (95)	5/18 (28)	7/15 (47)	-	22/34 (65)	2/3 (76)	3/34 (9)	9/34 (26)	5/34* (15)	2/34* (6)
**Overall***	*n = 261*	250/261 (96)	181/193 (94)	113/171 (66)	22/149 (15)	6/101 (6)	56/193 (29)	33/171 (19)	163/208 (78)	121/198 (61)	110/178 (62)	21/153 (14)	32/119 (27)	8/65 (12)	85/138 (62)	8/49 (16)	10/102 (10)	29/117 (25)	22/133 (17)	16/106 (15)

* = unsure how many screened

** = includes intracranial bleeds

*** = includes aortic aneurysm, borderline enlarged aortic root, aortic dissection

**** = data obtained from (17-46)

References

1 Gallione CJ, Repetto GM, Legius E, Rustgi AK, Schelley SL, Tejpar S, et al. A combined syndrome of juvenile polyposis and hereditary haemorrhagic telangiectasia associated with mutations in MADH4 (SMAD4). Lancet. 2004;363(9412):852-9.
2 Gallione CJ, Richards JA, Letteboer TG, Rushlow D, Prigoda NL, Leedom TP, et al. SMAD4 mutations found in unselected HHT patients. J Med Genet. 2006;43(10):793-7.
3 Poletto ED, Trinh AM, Levin TL, Tsirilakis K, Loizides AM. Hereditary hemorrhagic telangiectasia and juvenile polyposis: an overlap of syndromes. Pediatr Radiol. 2010;40(7):1274-7.
4 Gallione C, Aylsworth AS, Beis J, Berk T, Bernhardt B, Clark RD, et al. Overlapping spectra of SMAD4 mutations in juvenile polyposis (JP) and JP-HHT syndrome. Am J Med Genet A. 2010;152A(2):333-9.
5 Andrabi S, Bekheirnia MR, Robbins-Furman P, Lewis RA, Prior TW, Potocki L. SMAD4 mutation segregating in a family with juvenile polyposis, aortopathy, and mitral valve dysfunction. Am J Med Genet A. 2011;155A(5):1165-9.
6 Schwenter F, Ratjen F, Berk T, Gallinger S, Gryfe R, Gradinger AB, et al. Juvenile polyposis syndrome, SMAD4 mutations, and hereditary hemorrhagic telangiectasia. J Pediatr Gastroenterol Nutr. 2012;54(1):120-2.
7 Schwenter F, Faughnan ME, Gradinger AB, Berk T, Gryfe R, Pollett A, et al. Juvenile polyposis, hereditary hemorrhagic telangiectasia, and early onset colorectal cancer in patients with SMAD4 mutation. J Gastroenterol. 2012;47(7):795-804.
8 O'Malley M, LaGuardia L, Kalady MF, Parambil J, Heald B, Eng C, et al. The prevalence of hereditary hemorrhagic telangiectasia in juvenile polyposis syndrome. Dis Colon Rectum. 2012;55(8):886-92.
9 Wain KE, Ellingson MS, McDonald J, Gammon A, Roberts M, Pichurin P, et al. Appreciating the broad clinical features of SMAD4 mutation carriers: a multicenter chart review. Genet Med. 2014;16(8):588-93.
10 Heald B, Rigelsky C, Moran R, LaGuardia L, O'Malley M, Burke CA, et al. Prevalence of thoracic aortopathy in patients with juvenile Polyposis Syndrome-Hereditary Hemorrhagic Telangiectasia due to SMAD4. Am J Med Genet A. 2015;167A(8):1758-62.
11 Aytac E, Sulu B, Heald B, O'Malley M, LaGuardia L, Remzi FH, et al. Genotype-defined cancer risk in juvenile polyposis syndrome. Br J Surg. 2015;102(1):114-8.
12 de Leon MP, Pedroni M, Viel A, Luppi C, Conigliaro R, Domati F, et al. Massive juvenile polyposis of the stomach in a family with SMAD4 gene mutation. Fam Cancer. 2019;18(2):165-72.
13 Kilian A, Latino GA, White AJ, Clark D, Chakinala MM, Ratjen F, et al. Genotype-Phenotype Correlations in Children with HHT. J Clin Med. 2020;9(9).
14 McDonald NM, Ramos GP, Sweetser S. SMAD4 mutation and the combined juvenile polyposis and hereditary hemorrhage telangiectasia syndrome: a single center experience. Int J Colorectal Dis. 2020;35(10):1963-5.
15 Jelsig AM, Kjeldsen A, Christensen LL, Bertelsen B, Karstensen JG, Brusgaard K, et al. Hereditary haemorrhagic telangiectasia in Danish patients with pathogenic variants in SMAD4: a nationwide study. J Med Genet. 2022.
16 Kananazawa Y, Yamada T, Yamaguchi T, Saito Y, Kakinuma D, Masuda Y, et al. A novel germline SMAD4 variant detected in a Japanese family with juvenile polyposis syndrome and hereditary hemorrhagic telangiectasia. Jpn J Clin Oncol. 2022.
17 Sweet K, Willis J, Zhou XP, Gallione C, Sawada T, Alhopuro P, et al. Molecular classification of patients with unexplained hamartomatous and hyperplastic polyposis. JAMA. 2005;294(19):2465-73.
18 Iyer NK, Burke CA, Leach BH, Parambil JG. SMAD4 mutation and the combined syndrome of juvenile polyposis syndrome and hereditary haemorrhagic telangiectasia. Thorax. 2010;65(8):745-6.
19 Zimmer V, Grobholz R, Lammert F, Raedle J. Endoscopic resection of a large duodenal hamartoma related to SMAD4-associated juvenile polyposis/hereditary hemorrhagic teleangiectasia syndrome. Am J Gastroenterol. 2011;106(11):2047-8.
20 Teekakirikul P, Milewicz DM, Miller DT, Lacro RV, Regalado ES, Rosales AM, et al. Thoracic aortic disease in two patients with juvenile polyposis syndrome and SMAD4 mutations. Am J Med Genet A. 2013;161A(1):185-91.
21 Sartor C, Papayannidis C, Chiara Abbenante M, Iacobucci I, Broccoli A, Venturi C, et al. Recurrent Gastrointestinal Hemorrhage in Treatment with Dasatinib in a Patient Showing SMAD4 Mutation with Acute Lymphoblastic Leukemia Philadelphia Positive and Juvenile Polyposis Hereditary Hemorrhagic Telangiectasia Syndrome. Hematol Rep. 2013;5(2):26-7.
22 Anderson B, Baron TH, Sweetser S. A woman with melena and transfusion-dependent anemia. Gastroenterology. 2014;146(3):621-873.
23 Mazzucco S, Benini L, Gallione C, D'Adamo P, Girelli D. Juvenile stroke in combined syndrome of hereditary hemorrhagic telangiectasia and juvenile polyposis. Neurol Sci. 2014;35(8):1315-8.
24 Lin HC, Fiorino KN, Blick C, Anupindi SA. A rare presentation and diagnosis of juvenile polyposis syndrome and hereditary hemorrhagic telangiectasia overlap syndrome. Clin Imaging. 2015;39(2):321-4.
25 Johansson J, Sahin C, Pestoff R, Ignatova S, Forsberg P, Edsjo A, et al. A Novel SMAD4 Mutation Causing Severe Juvenile Polyposis Syndrome with Protein Losing Enteropathy, Immunodeficiency, and Hereditary Haemorrhagic Telangiectasia. Case Reports in Gastrointestinal Medicine. 2015;2015.
26 Kadiyska T, Nossikoff A, Kratunkov P, Hachmerian M, Angelova L. Clinical and genetic challenges in a family with history of childhood polyp, aortopathy, and clinical diagnosis of hereditary hemorrhagic teleangiectasia (HHT). Ann Pediatr Cardiol. 2016;9(2):176-8.
27 Ramos GP, Sharain K, Ravi K. Juvenile polyposis and hereditary hemorrhagic telangiectasia overlap syndrome. Gastrointest Endosc. 2017;86(1):238-9.
28 Bishop JC, Britton JF, Murphy AM, Sule S, Mitchell S, Takemoto C, et al. Juvenile Idiopathic Arthritis Associated with Combined JP-HHT Syndrome: A Novel Phenotype Associated with a Novel Variant in SMAD4. J Pediatr Genet. 2018;7(2):78-82.
29 Inoguchi Y, Kaku B, Kitagawa N, Katsuda S. Hereditary Hemorrhagic Telangiectasia with SMAD4 Mutations Is Associated with Fatty Degeneration of the Left Ventricle, Coronary Artery Aneurysm, and Abdominal Aortic Aneurysm. Intern Med. 2019;58(3):387-93.
30 Karlsson T, Cherif H. Mutations in the ENG, ACVRL1, and SMAD4 genes and clinical manifestations of hereditary haemorrhagic telangiectasia: experience from the Center for Osler's Disease, Uppsala University Hospital. Ups J Med Sci. 2018;123(3):153-7.
31 Chung AD, Mortele KJ. Combined juvenile polyposis syndrome and hereditary hemorrhagic telangiectasia (JPS/HHT) with MRI and endoscopic correlation. Clin Imaging. 2019;54:37-9.
32 Hashimoto Y, Yokoyama K, Kumagai H, Okada Y, Yamagata T. Juvenile polyposis syndrome-hereditary hemorrhagic telangiectasia associated with a SMAD4 mutation in a girl. Clin J Gastroenterol. 2020;13(6):1096-101.
33 Chang W, Renaut P, Pretorius C. SMAD4 juvenile polyposis syndrome and hereditary haemorrhagic telangiectasia presenting in a middle-aged man as a large fungating gastric mass, polyposis in both upper and lower GI tract and iron deficiency anaemia, with no known family history. BMJ Case Rep. 2020;13(12).
34 Kang B, Hwang SK, Choi S, Kim ES, Lee SY, Ki CS, et al. Case report of juvenile polyposis/hereditary hemorrhagic telangiectasia syndrome: first report in Korea with a novel mutation in the SMAD4 gene. Transl Pediatr. 2021;10(5):1369-76.
35 Mrzljak A, Popic J, Ozanic Bulic S. Digital Clubbing in Hereditary Hemorrhagic Telangiectasia/Juvenile Polyposis Syndrome. Acta Dermatovenerol Croat. 2021;291(1):56-7.
36 Leonard NB, Bronner MP. Giant Gastric Folds in Juvenile Polyposis. Case Rep Gastroenterol. 2021;15(3):985-93.
37 Gheewalla GM, Luther J, Das S, Kreher JB, Scimone ER, Wong AW, et al. An additional patient with SMAD4-Juvenile Polyposis-Hereditary hemorrhagic telangiectasia and connective tissue abnormalities: SMAD4 loss-of-function and gain-of-function pathogenic variants result in contrasting phenotypes. Am J Med Genet A. 2022;188(10):3084-8.
38 Dal Buono A, Poliani L, Repici A, Hassan C, Bianchi P. An Unexpected Anemia Hiding a Rare Syndrome With Overlapping Phenotypes. ACG Case Rep J. 2022;9(11):e00926.
39 Tao MY, Wang KY, Li X, Yu C, Wan QS, Shu X, et al. Hereditary hemorrhagic telangiectasis with juvenile polyposis syndrome: a case report. Therap Adv Gastroenterol. 2022;15:17562848221142913.
40 Korzenik J, Chung DC, Digumarthy S, Badizadegan K, Moss JD, Harris NL, et al. A 43-year-old man with lower gastrointestinal bleeding - Hereditary hemorrhagic telangiectasia and atypical juvenile polyposis with dysplasia and intramucosal carcinoma, associated with a germ-line mutation in the MADH4 gene. New England Journal of Medicine. 2005;353(17):1836-44.
41 Nishida T, Faughnan ME, Krings T, Chakinala M, Gossage JR, Young WL, et al. Brain arteriovenous malformations associated with hereditary hemorrhagic telangiectasia: Gene-phenotype correlations. American Journal of Medical Genetics Part A. 2012;158A(11):2829-34.
42 Oliveira MM, Meloni VA, Canonaco RS, Takeno SS, Bortolai A, de Mello CB, et al. Juvenile Polyposis/Hereditary Hemorrhagic Telangiectasia Syndrome in an Adolescent With Complex Chromosomal Rearrangement and Intellectual Disability. American Journal of Medical Genetics Part A. 2014;164(10):2685-8.
43 Rossetti L, Cornelius A. SMAD4-related juvenile polyposis/hereditary hemorrhagic telangiectasia syndrome presenting as metastatic adenocarcinoma of the colon in a teenager - A case report. Genetics in Medicine. 2022;24(3):S139-S.
44 Shikata K, Kukita Y, Matsumoto T, Esaki M, Yao T, Mochizuki Y, et al. Gastric juvenile polyposis associated with germline SMAD4 mutation. American Journal of Medical Genetics Part A. 2005;134A(3):326-9.
45 Wiener E, Martin P, Mehta S, Markus HS. CERVICAL ARTERY DISSECTION AND ILIAC ARTERY ANEURYSM IN AN SMAD-4 MUTATION CARRIER. Neurology-Genetics. 2017;3(5).
46 Bruceta M, De Souza L, Carr Z, Bonavia A, Karamchandani K. Novel Association of Juvenile Polyposis Syndrome With Atrial Septal Aneurysm and Patent Foramen Ovale: A Case Report. A A Pract. 2018;10(12):331-4.
